# Effects of cold water immersion after exercise on fatigue recovery and exercise performance--meta analysis

**DOI:** 10.3389/fphys.2023.1006512

**Published:** 2023-01-20

**Authors:** Feiyan Xiao, Anastasiia V. Kabachkova, Lu Jiao, Huan Zhao, Leonid V. Kapilevich

**Affiliations:** ^1^ Faculty of Physical Education, Tomsk State University, Tomsk, Russia; ^2^ Sports Coaching College, Beijing Sport University, Beijing, China; ^3^ Central Research Laboratory, Siberian State Medical University, Tomsk, Russia

**Keywords:** delayed-onset muscle soreness, cold water immersion, exercise fatigue, exercise-induced soreness, fatigue recovery measures

## Abstract

Cold water immersion (CWI) is very popular as a method reducing post-exercise muscle stiffness, eliminating fatigue, decreasing exercise-induced muscle damage (EIMD), and recovering sports performance. However, there are conflicting opinions as to whether CWI functions positively or negatively. The mechanisms of CWI are still not clear. In this systematic review, we used meta-analysis aims to examine the effect of CWI on fatigue recovery after high-intensity exercise and exercise performance. A total of 20 studies were retrieved and included from PubMed, PEDro and Elsevier databases in this review. Publication years of articles ranged from 2002 to 2022. In selected studies including randomized controlled trials (RCTs) and Crossover design (COD). Analyses of subjective indicators such as delayed-onset muscle soreness (DOMS) and ratings of perceived exertion (RPE), and objective indicators such as countermovement jump (CMJ) and blood plasma markers including creatine kinase(CK), lactate/lactate dehydrogenase(LDH), C-reactive protein(CRP), and IL-6 were performed. Pooled data showed as follows: CWI resulted in a significant decline in subjective characteristics (delayed-onset muscle soreness and perceived exertion at 0 h); CWI reduced countermovement jump(CMJ) significantly at 0 h, creatine kinase(CK) was lowered at 24 h, and lactate at 24 and 48 h. There was no evidence that CWI affects C-reactive protein(CRP) and IL-6 during a 48-h recovery period. Subgroup analysis revealed that different CWI sites and water temperatures have no effect on post-exercise fatigue recovery. Recommended athletes immersed in cold water immediately after exercise, which can effectively reduce muscle soreness and accelerate fatigue recovery.

## Introduction

Physiological disorders such as muscle damage, hyperthermia, dehydration, and glycogen depletion can occur after high-intensity training. Insufficient or unreasonable relaxation after long-term intensive training can adversely affect the subsequent training performance or competitions (Spencer et al., 2005; Rowsell et al., 2009). Fatigue can build up after unreasonable relaxation, which affects athletes’ physical health and decreased exercise performance (Gill et al., 2006). Exercise, training, or competing will not be affected after exercise if there is sufficient recovery time. However, when the post-exercise rest period is very short, the problem of reducing or eliminating fatigue and muscle damage, and maintaining or improving performance for the next exercise becomes very urgent.

In an attempt to alleviate fatigue and damage caused by exercise, it is imperative to fully warm up before training or competing, standardize movements during the process, and fully relax in time afterwards. Additionally, multiple external intervention methods have been adopted for athletes, including myoelectric stimulation, anti-inflammatory drugs, antioxidants, and cold water immersion(CWI) (Barnett, 2006). Among them, CWI is currently more popular in training and competing. Studies have shown that CWI reduces post-exercise muscle stiffness, eliminates fatigue, decreases exercise-induced muscle damage (EIMD), and improves athletic performance. Furthermore, athletes can accomplish more work in subsequent exercise after CWI (Roberts et al., 2014). Other evidence indicates that CWI negatively affects exercise performance (Paddon-Jones & Quigley, 1997; Sellwood et al., 2007), which may just be a placebo effect (Broatch et al., 2014; White et al., 2014). To determine the physiological mechanisms of CWI intervention and the possibility of it as an external relaxation tool during sports competitions, the effects of CWI intervention on sports fatigue and injury, as well as exercise performance after high-intensity exercise, were examined in this review.

## Methods

### Literature search strategies

According to the PRISMA statement, this systematic review was conducted from September 2021 and March 2022 in the PubMed, PEDro and Elsevier databases (Moher et al., 2015). The following search terms were used to find the relevant articles: 1) cold water immersion(CWI) OR cooling OR ice bath; 2) exercise performance OR sports performance; 3) fatigue OR recovery. The language restriction (articles published in English only) was adopted. Publication years of articles ranged from 2002 to 2022. In fact, articles after 2018 have not been included in present study. First of all, articles were not relevant, the control group of article did not use passive rest but other interventions. Secondly, even if the article was relevant, the researchers failed to contact the author of the article to obtain relating data.

### Selection variables

**FIGURE 1:**
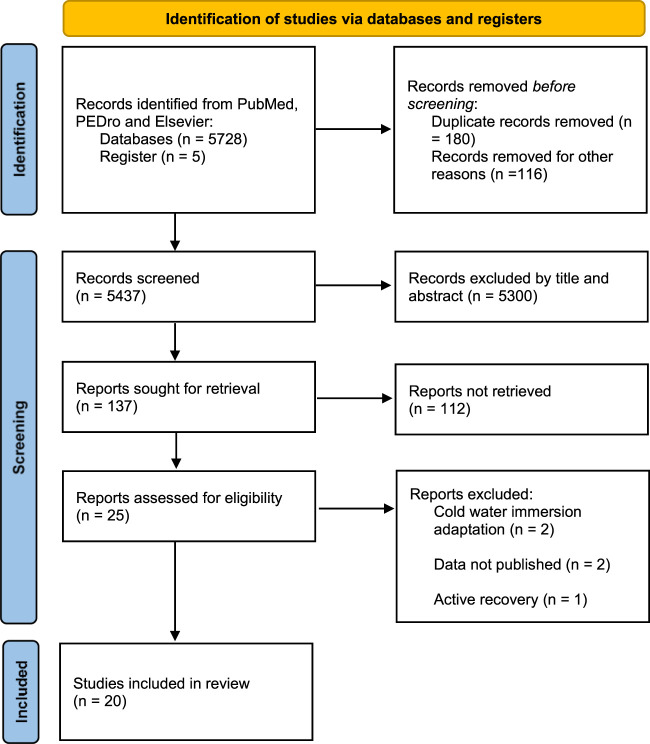
Flow-chart describing the systematic review procedure.

Muscle soreness and fatigue recovery status, which can be characterized by delayed-onset muscle soreness (DOMS) and ratings of perceived exertion (RPE), are generally measured by subjective scales. Countermovement jump (CMJ) performance is considered an easy-to-implement and valuable indicator of muscle strength, a significant predictor of maximum speed and explosive force in sports, and a useful method for assessing mobility and functional capacity in fatigued athletes. The release of creatine kinase (CK), lactate, and lactate dehydrogenase (LDH) in plasma has been used as an indirect biochemical marker for exercise muscle injury to characterize muscle membrane disruption. IL-6 has traditionally been taken as a marker of post-exercise inflammation. C-reactive protein (CRP) is an indicator of infectious or inflammatory conditions (Clyne & Olshaker, 1999). Consequently, the indicators including DOMS, RPE, CMJ, CK, Lactate/LDH, IL-6, and CRP were included in this review to examine whether performing CWI after exercise can affect the above indicators, which were measured immediately(0 h) and/or 24 h and/or 48 h after exhaustive exercise. DOMS and RPE were defined as subjective recovery characteristics, and CMJ, CK, lactate/LDH, CRP, and IL-6 as objective recovery characteristics (Hohenauer et al., 2015). The systematic search strategy and process are shown in Figure 1.

### Study inclusion and exclusion criteria

The present selected studies must include human subjects who received CWI intervention after exercise. CWI is defined as immersion in water at ≤15°C (Wilcock et al., 2006). In selected studies including randomized controlled trials (RCTs) and Crossover design (COD), the effect of post-exercise CWI on subsequent muscle soreness was examined. The inclusion criteria for studies were as follows: 1) RCTs and COD; 2) healthy humans; 3) at least one of the two outcome variables reported; 4) CWI performance at ≤15°C; 5) having performed baseline and post-training assessments of fatigue/soreness indicators; 6) the human volunteers could be of any athletic training status; 7) no sex-specific inclusion or exclusion criteria. Studies were excluded in the following cases: 1) not scrupulous experimental design; 2) duplicate publication; 3) animal experiments; 4) English language restrictions.

### Data analysis

Meta-analysis calculations were performed by Revman 5.4 software. Two researchers (JL and ZH) independently extracted the general study features and specific study results. The disagreement between the two authors (JL and ZH) was resolved by consensus or third-party adjudication. The outcome indicators in the included literature were continuous variables. The mean difference (MD) was selected as the effect scale indicator for statistical analysis when the unit was unified, and standardized mean difference (SMD) was chosen when the unit or method for measurement was different. The I^2^ statistic was used to test the heterogeneity among the studies. There was no heterogeneity among studies when I^2^ = 0; there was heterogeneity among studies when I^2^ > 50%. Fixed-effects models were applied in case of no heterogeneity; random-effects models were implemented in case of heterogeneity. Subgroup and sensitivity analyses were performed if heterogeneity was observed, or meta-analyses were repeated after excluding studies with abnormal results. The publication bias analysis was performed by a funnel plot.

## Risk of bias

Cochrane’s risk-of-bias tool was adopted to independently assess all included articles by two authors (Higgins et al., 2019). Each article was scored in the following aspects: random sequence generation, allocation concealment, blinding participants, blinding personnel, blinding outcome assessors, incomplete outcome data, and other sources of bias. They were rated ‘low’ (+) if the criteria for items at low risk of bias were met, or ‘high’ (-) if the criteria for items at high risk of bias were met. Additionally, risk of bias items were deemed “unclear” if insufficient information in the article or material led to doubtful interpretation (e.g. no symbols for indication).

### Subgroup analysis

Subgroup analyses were conducted based on CWI of different body parts (shoulder, umbilicus), and the water temperature was <10°C or ≥10°C in performing CWI.

## Results

### Risk of bias

**FIGURE 2:**
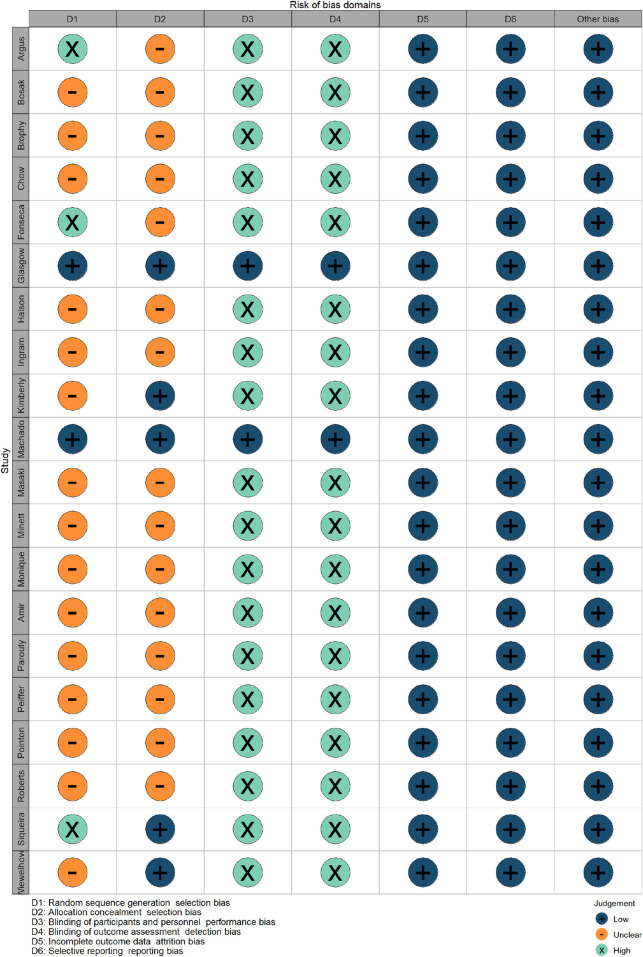
Risk of bias graph for all included studies.

**FIGURE 3:**
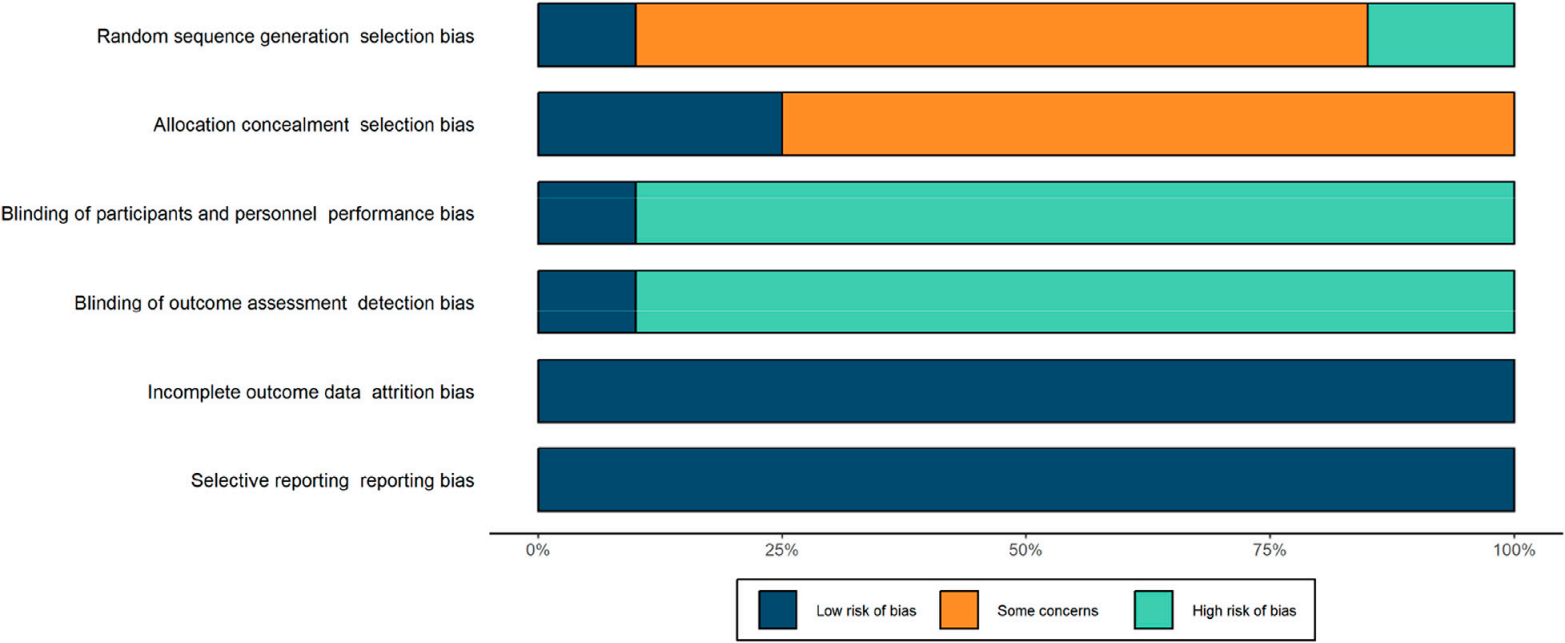
Risk of bias summary for all included studies.

Blinding of participants and personnel, and blinding of outcome assessment demonstrated a high risk, whereas allocation concealment demonstrated an unclear risk. Random allocation of participants was used only in five studies (Rupp et al., 2012; Glasgow et al., 2014; Machado et al., 2016; Siqueira et al., 2018). Blinding of the participants and blinding of outcome assessment were only found in two studies (Glasgow et al., 2014; Machado et al., 2016). Detailed information about the risk of bias is shown in Figures 2, 3.

### Research characteristics

The characteristics of the selected studies are shown in Table 1. A total of 20 studies were included in the analysis (Bosak et al., 2006; Halson et al., 2008; Ingram et al., 2009; Peiffer et al., 2010a; Parouty et al., 2010; Brophy-Williams et al., 2011; Pointon et al., 2012a; Pointon and Duffield, 2012; Rupp et al., 2012; Glasgow et al., 2014; Minett et al., 2014; Roberts et al., 2014; Takeda et al., 2014; Fonseca et al., 2016; Machado et al., 2016; Amir et al., 2017; Argus et al., 2017; Chow et al., 2018; Siqueira et al., 2018; Wiewelhove et al., 2018). And the trials were used as a primary data source for this review. The meta-analysis was adopted in the introduction and conclusion sections. There were a total of n = 419 healthy subjects including males (n = 360) and females (n = 59) in the selected articles, whereas only n = 357 healthy participants were involved in the experiments.

**TABLE 1:** Summary of the included studies.

Study,year	Characteristics of participants (training status, sex(m:f), age)	Enviroment condition(Tm ± S;RHm ± S)	Exercise protocol	Classification of the exercise	[CWI duration and temperature]×number	Control group	Outcome variables and time of measurement post exercise(h)
Bosak et al., 2006(Bosak et al., 2006)	Regularly runners (9:3); 21 ± 1 year	19°C ± 2°C	5 km trial	High intensity	12 min Ice water immersion	24 h passive recovery	PRE (0)
Jonathan Parouty et al., 2010(Parouty et al., 2010)	well-trained swimmers(5:5); 19.0 ± 3.9 years		Maximal 100-m freestyle swimming sprints	High intensity	[5min at 14°C–15°C]×1	[5min at 28°C air]×1	Lactate (0) PRE (0)
Peiffer et al., 2010(Peiffer et al., 2010a)	Cyclists(10:0); 29 ± 6 years	35.0°C ± 0.3°C; 40.0 ± 3.0%RH	1-km time trial as fast as possible	High intensity	[15min at 35°C air+5min at 14°C]×1	[20min at 35°C air]×1	PRE (0)
Brophy-Williams et al., 2011(Brophy-Williams et al., 2011)	Well trained athletes(8:0); 20.9 ± 1.2 years	16.3°C ± 1.5°C; 50.1 ± 5.9%RH	Yoyo Intermittent Recovery Test	High intensity	15min at 15°C ± 1°C]×1	[15min at 23°C]×1	Lactate (0)
Rupp et al., 2012(Rupp et al., 2012)	Collgiate soccer players(13:9); 19.8 ± 1.1 yrs	6.1°C; 62%RH	Yoyo Intermittent Recovery Test	High intensity	15min at 12°C	15min at room temperature	CMJ(0;24;48); DOMS(0;24;48)
Chow et al., 2018(Chow et al., 2018)	Amateur rugby players(29:24); 21.6 ± 2.9 years	25̊C; 75%RH	[submaximal movement 5s]×5 +[15s sprint]×14	High intensity	1min at 5°C	1 min passive rest	CMJ(0)
Fonseca et al., 2016(Fonseca et al., 2016)	Highly trained athletes(8:0); 24 ± 3.6 years		120 min jiu-jitsu(40min warmup+40min technical training+40min combat simulation)	High-intensity	16min at 6.0°C	Passive rest	CK(0;24;48); LDH(0;24;48); CMJ(0;24;48)
Pointon et al., 2012(Pointon et al., 2012a)	Rugby athletes(10:0); 19.9 ± 1.1 yrs	32°C; 52% RH	2 × 30 min intermittent-sprint exercise (ISE)	high-intensity	20min at8.9°C ± 0.9°C	20min Passive rest	Lactate(0); CK(0;24); CRP(0;24)
Roberts et al., 2014(Roberts et al., 2014)	physically active man(10:0); 21.3 ± 1.6 years	24.3°C ± 0.6°C; 48.6 ± 1.2%RH	1 h resistance training	High-intensity	10 min at 10°C ± 0.3°C	10min active recovery	CMJ(0)
Wiewelhove et al., 2018(Wiewelhove et al., 2018)	Recreational runners(46:0); 30.5 ± 10.9		Half- marathon	High-intensity	15min at 15°C ± 1°C	15min passive recovery	CMJ(0;24); DOMS(0;24); CK(0;24); CRP(0;24)
Minett et al., 2014(Minett et al., 2014)	Moderate-to well-trained male(9:0); 21 ± 2years	32.4°C ± 1.0°C; 42.4 ± 6.1% RH	2 × 35 min intermittent-sprint exercise	High-intensity	20min at 10.0 °C± 0.4°C cold water	20min passive recovery at 32°C and 42% RH	CK(0;24); CRP(0;24)
Pointon et al., 2012(Pointon and Duffield, 2012)	Well-trained rugby athletes(10:0); 21.0 ± 1.7 years	20.3°C ± 1.1°C; 37.0% ± 1.1%RH	2 × 30 min Intermittent-sprint exercise	High-intensity	2 × 9 min at 9.2°C ± 0.2°C followed by 1 min seat in room	20 min passive recovery	Lactate(0); CK(0); CRP(0;24)
Argus et al., 2017(Argus et al., 2017)	males (13:0); 26 ± 5 years		50 min resistance training protocol	High-intensity	14min at 15°C	14min passive recovery	DOMS(0); VAS(0)
Masaki Takega et al., 2014(Takeda et al., 2014)	Well-trained collegiate male rugby players (20:0); 20.3 ± 0.6 years		80 min of rugby game simulation training	High-intensity	10min at 15°C	15min passive recovery	CMJ(24); RPE(0;24) СK(24); Lactate(24) LDH(24)
N.H. Amir et al., 2017(Amir et al., 2017)	Physically healthy young males(16:0); 21.6 ± 2.3 years		Plyometric exercise protocol	High-intensity	15min at 15 °C± 1°C	15min passive recovery	VAS(24;48); СK(24;48); LDH(24;48)
Siqueira et al., 2018(Siqueira et al., 2018)	physically active males(30:0) Con:19.9 ± 1.4years CWI:20.5 ± 1.4 years		2×(20 times drop from 60-cm box)	High-intensity	20min at 10°C ± 1°C	20min passive recovery	CK(24); CRP(24)
Machado et al., 2017(Machado et al., 2017)	Healthy male(60:0); 18–25 years	21°C–23°C; 40%–60% RH	5×(15 times eccentric contraction of knee extension, separated by 30s)	High-intensity	CWI9: 15min at 9°C; CWI14: 15min at 14°C	15min passive recovery	CK(24;48;72); VAS(24;48;72); RPE(24;28;72)
Glasgow et al., 2014(Glasgow et al., 2014)	Healthy subjects(32:18); 18–35 years		Eccentric hamstring contractions to fatigue	High-intensity	CWI6: 10min at 6°C; CWI10: 10min at 10°C	10min passive recovery	CK(24;48;72); VAS(24;48;72)
Halson et al., 2008(Halson et al., 2008)	Endurance-trained cyclists(11:0); 23.8 ± 1.6 years	34.3°C ± 1.1°C; 41.2 ± 3.0%RH	40 min cycling	High-intensity	3 × 60 S separated by 120 S at 11.5°C ± 0.3°C	20min passive recovery	CK(0); CRP(0); IL-6(0)
Ingram et al., 2009(Ingram et al., 2009)	Athletes; (11:0); 27.5 ± 6.0 years		80 min simulated team sports+20 min shuttle run test to exaustion	High-intensity	5 × 2min separated by 2.5min at 10°C	15min passive recovery	DOMS(0;24;48); CK(0;24;48); CRP(0;24;48)

### Characteristics of exercise protocols

The exercise protocols included football matches, rugby match simulations (Takeda et al., 2014), cycling (Halson et al., 2008; Peiffer et al., 2010a), swimming (Parouty et al., 2010), jumping (Amir et al., 2017; Siqueira et al., 2018), and running (Bosak et al., 2006; Ingram et al., 2009; Pointon et al., 2012a; Minett et al., 2014; Takeda et al., 2014; Chow et al., 2018; Wiewelhove et al., 2018). All running protocols were endurance running ones. Two authors adopted the YoYo intermittent recovery test (Brophy-Williams et al., 2011; Rupp et al., 2012). In one study, jiu-jitsu was used to ensure the pre-cooling load (Fonseca et al., 2016). In a total of two studies, the functional exercise was chosen (Roberts et al., 2014; Argus et al., 2017). The eccentric contraction was adopted in two articles (Glasgow et al., 2014; Machado et al., 2017). A detailed overview of the performed exercise protocols is shown in Table 1.

### Characteristics of CWI

In the present included studies, the most common body parts of CWI were submerged to the iliac crest, the legs immersed, to ensure that the lower limbs were fully submerged in the tubs. The water temperature for immersion to the iliac crest ranged from 5°C to 15°C (Pointon et al., 2012a; Pointon and Duffield, 2012; Machado et al., 2016; Amir et al., 2017; Chow et al., 2018; Siqueira et al., 2018; Wiewelhove et al., 2018), including the water temperature for immersion to the umbilicus (Ingram et al., 2009; Rupp et al., 2012). In the other studies, it was ensured that participants were submerged in the water bath, with the water immersion to the mid-sternal level (Halson et al., 2008; Peiffer et al., 2010a; Brophy-Williams et al., 2011; Glasgow et al., 2014; Minett et al., 2014) and shoulders (Parouty et al., 2010; Roberts et al., 2014; Takeda et al., 2014; Fonseca et al., 2016; Argus et al., 2017). The water temperature was 6°C–15°C in both mid-sternal and shoulder immersion studies.

### Characteristics of the passive Control group

Common passive CONs were sitting on a chair in a room with a normal temperature between 6°C and 32°C, and a relative humidity between 37% and 62%. Active cyclists were taken as the CON group because athletes usually perform low-intensity warm-ups to maintain their body temperature instead of passively waiting when resting between competitions (Roberts et al., 2014). It was decided that this article was not excluded in this review, because active recovery was chosen over passive recovery in it. Due to the low intensity of the active exercise, this interference could be negligible (subjects can self-select the intensity on an ergometer).

#### CMJ

**FIGURE 4:**
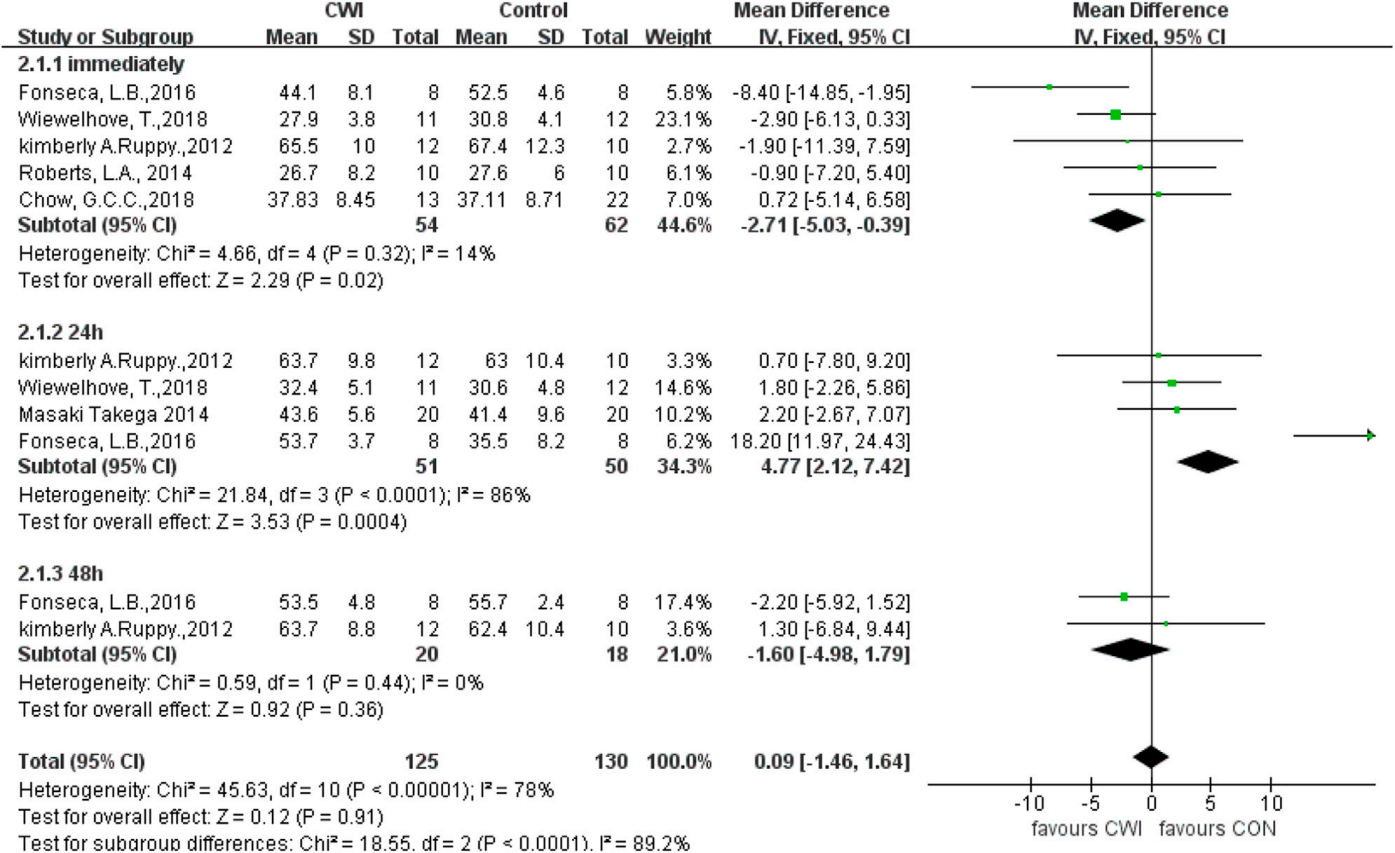
Forest plot of the comparison of CWI versus CON for measurement of CMJ. CWI = Cold water immersion, CON = Control, CMJ = countermovement jump.

Current studies indicate that CMJ immediately and 24 h after CWI are significantly different from that in the CON group (immediately: MD -2.71, 95%CL -5.03 to -0.39, 5 trials); (24 h: MD 4.77, 95%CL 2.12 to 7.42, 4 trials); but there was no significant different between the CWI and CON groups at 48 h (MD -1.60, 95%CL -4.98 to 1.79, two trials) (Figure 4). However, there was significant heterogeneity in the data at 24 h (I^2^ = 86%), so a random effects model was used. At this point, there was no significant difference in the CMJ between the CWI and CON groups at 24 h (SMD 5.72, 95%CL -1.83 to 13.27, 4 trials). And the CMJ results after the intervention were unchanged from those at 0 and 48 h, respectively.

A sensitivity analysis was performed to investigate whether heterogeneity among studies was caused by individual studies. At 24h, heterogeneity decreased after excluding the study by Fonseca, L, B (Chi^2^ = 0.09, df = 2 (*p* = 0.96), I^2^ = 0%), showing that this study was the source of heterogeneity, but with the effect size unchanging significantly at this time (MD 1.81 95%CL -1.11 to 4.74, 3 trails), suggesting stable study results.

Subgroup analysis was performed on potential moderator variables to further explore the sources of heterogeneity in CMJ at 24 h. The groups were divided into the navel and shoulder immersion ones according to CWI of different body parts, and subgroup analysis was performed, which revealed heterogeneity in the shoulder immersion group at 24 h (Chi^2^ = 15.71, df = 1 (*p* < 0.0001), I^2^ = 94%). And hence a random effects model was used, and the results showed no significant difference. There was no heterogeneity in the umbilicus immersion group at 24 h (Chi^2^ = 0.05, df = 1 (*p* = 0.82), I^2^ = 0%), so a fixed-effects model was used, and the results showed no significant differences. And there was no significant difference between the two subgroups of CWI to the shoulder and the umbilicus (Test for subgroup differences: Chi^2^ = 1.07, df = 1(*p* = 0.30), I^2^ = 6.2%), indicating that CWI of different body parts was not a source of heterogeneity and that CWI to different sites was ineffective in improving CMJ performance at 24 h after exercise.

CWI participants were divided into two groups at <10°C and ≥10°C, and subgroup analysis revealed significant differences between the two groups at 24 h (Test for subgroup differences: Chi^2^ = 21.75, df = 1 (*p* < 0.00001), I^2^ = 95.4%), suggesting that the difference in cold water temperature accounts for heterogeneity. Post-exercise immersion in cold water at a temperature <10°C can enhance CVJ at 24 h. However, since there was only 1 article involving a <10°C group, this may contribute to the uncertainty of the results.

#### DOMS

**FIGURE 5:**
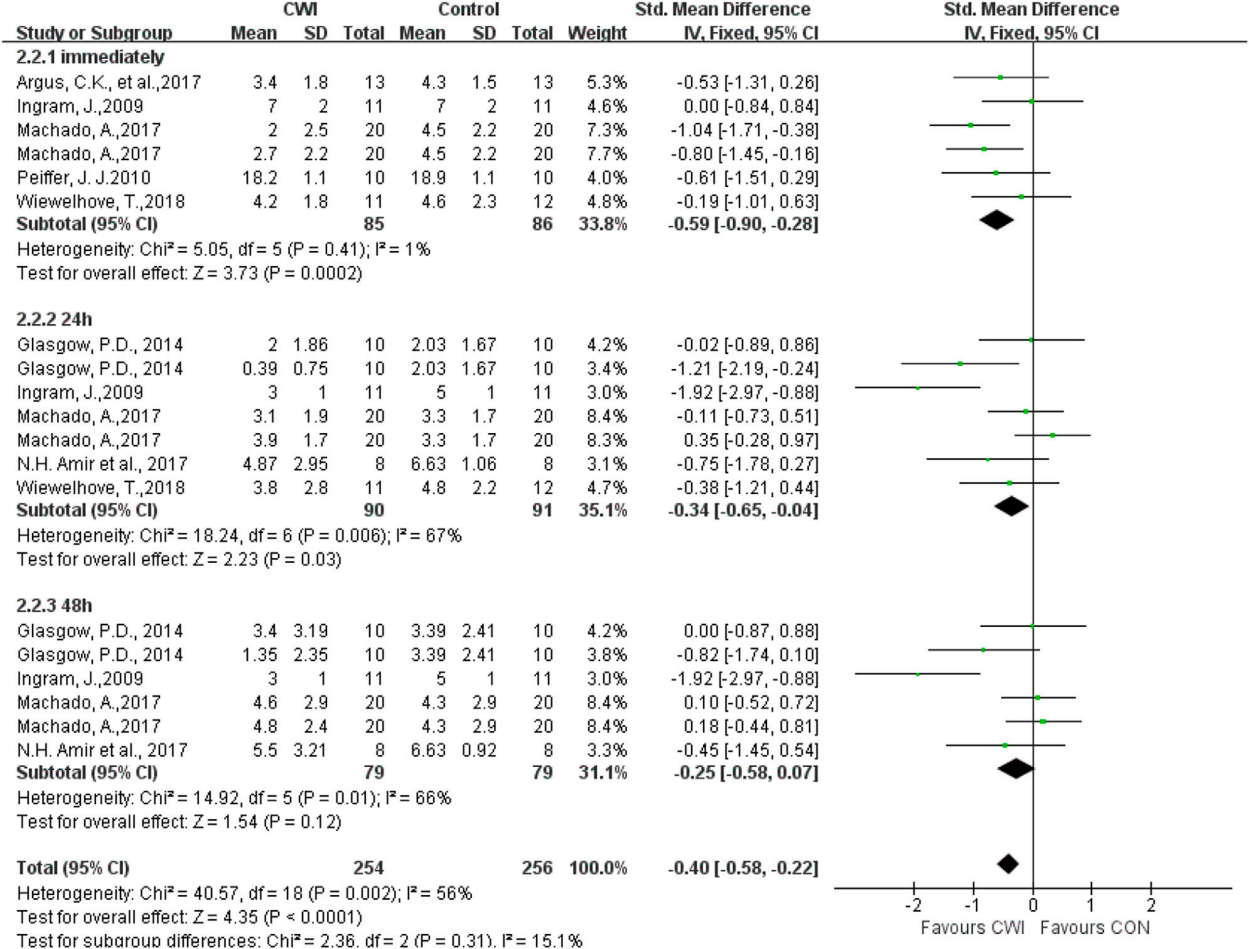
Forest plot of the comparison of CWI versus CON for measurement of DOMS. CWI = Cold water immersion, CON = Control, DOMS = delayed-onset muscle soreness.

Among the DOMS indicators, a total of seven articles were selected by different scales, visual analogue scale/score(VAS) 1–100 mm, 1–10 cm, 0–10 cm, and therefore SMD was chosen for analysis (Figure 5). The DOMS was measured 0h, 24h, and 48 h after the experimental intervention. The results showed that DOMS levels in the CWI group were significantly lower than those in the CON group at 0h, 24 h (0 h: SMD -0.59, 95%CL -0.90 to -0.28, 6 trials); (24 h: SMD -0.34, 95%CL -0.65 to -0.04, 7 trials), and no significant difference was found between the CWI and CON groups at 48 h (48 h: SMD -0.25, 95%CL -0.58 to 0.07, 6 trials). Heterogeneity was found between the literature at 24 and 48 h (24 h: I^2^ = 67%; 48 h: I^2^ = 66%), and hence the random effects model was chosen. The DOMS results were unchanged at 0h, and there was no longer a significant difference at 24 h (24 h: SMD -0.50, 95%CL -1.05 to 0.04, 7 trials); (48 h: SMD -0.41, 95%CL -0.98 to 0.71, 6 trials). The results showed that post-exercise CWI intervention was effective in reducing DOMS immediately, but not for prolonged periods of time (24 h, 48 h).

A sensitivity analysis was performed by the one-by-one exclusion of literature to investigate whether the heterogeneity between studies was caused by individual studies. The heterogeneity was found to decrease after excluding the study by Ingram et al., 2009 at 24 and 48 h (24 h: Chi^2^ = 8.66, df = 5 (*p* = 0.12), I^2^ = 42%); (48 h: Chi^2^ = 4.08, df = 4(*p* = 0.40), I^2^ = 2%), showing that this study was the source of heterogeneity. Nevertheless, the effect size did not change significantly (24 h: SMD -0.26 95%CL -0.69 to 0.16, 6 trails); (48 h: SMD -0.08 95%CL -0.42 to 0.26, 5 trails), suggesting stable study results.

CWI subgroup analysis was performed based on CWI of different body parts to further explore potential sources of heterogeneity. Heterogeneity was observed in the shoulder immersion group at 24 h (Chi^2^ = 3.21, df = 1(*p* = 0.07), I^2^ = 69%). A random effects model was used and there was no significant difference in results. There was no heterogeneity at 48 h (Chi^2^ = 1.61, df = 1(*p* = 0.20), I^2^ = 38%), and the results were not significantly different when a fixed effects model was used, indicating that post-exercise CWI to the shoulder was ineffective in changing muscle soreness at 24 and 48 h. Heterogeneity also existed in the umbilicus immersion group at 24 and 48 h (24 h: Chi^2^ = 14.52, df = 4(*p* = 0.006), I^2^ = 72%); (48 h: Chi^2^ = 13.08, df = 3(*p* = 0.004), I^2^ = 77%), and none of the differences were statistically significant when a random effects model was used, indicating that post-exercise CWI to the umbilicus was ineffective in altering muscle soreness at 24 or 48 h. There were no significant differences between the two subgroups of CWI to the shoulder and the umbilicus (Test for subgroup differences: 24 h: Chi^2^ = 0.03, df = 1 (*p* = 0.87), I^2^ = 0%; 48 h: Chi^2^ = 0.01, df = 1 (*p* = 0.94), I^2^ = 0%), indicating that the difference in CWI sites is not a factor in DOMS heterogeneity.

Subgroup analysis was performed based on the difference in CWI temperature, revealing heterogeneity in the <10°C group at 24 and 48 h (24 h: Chi^2^ = 3.53, df = 1(*p* = 0.06), I^2^ = 72%), (48 h: Chi^2^ = 2.65, df = 1(*p* = 0.10), I^2^ = 62%). A random effects model was used and no significant effect on the results was found. Heterogeneity was found in the ≥10°C group at 24 and 48 h (24 h: Chi^2^ = 14.57, df = 4(*p* = 0.006), I^2^ = 73%), (48 h: Chi^2^ = 12.16, df = 3(*p* = 0.007), I^2^ = 75%). Then, a random effects model was used to show no significant effect on the results. Also, it was discovered that at <10°C or ≥10°C, post-exercise CWI was both ineffective in changing DOMS at 24 and 48 h. In addition, there was no significant difference between the two subgroups at different CWI temperatures (Test for subgroup differences: 24 h: Chi^2^ = 0.03, df = 1 (*p* = 0.86), I^2^ = 0%; 48 h: Chi^2^ = 0.09, df = 1 (*p* = 0.76), I^2^ = 0%). Therefore, the changes in CWI temperature do not contribute to heterogeneity between DOMS groups.

#### RPE

**FIGURE 6:**
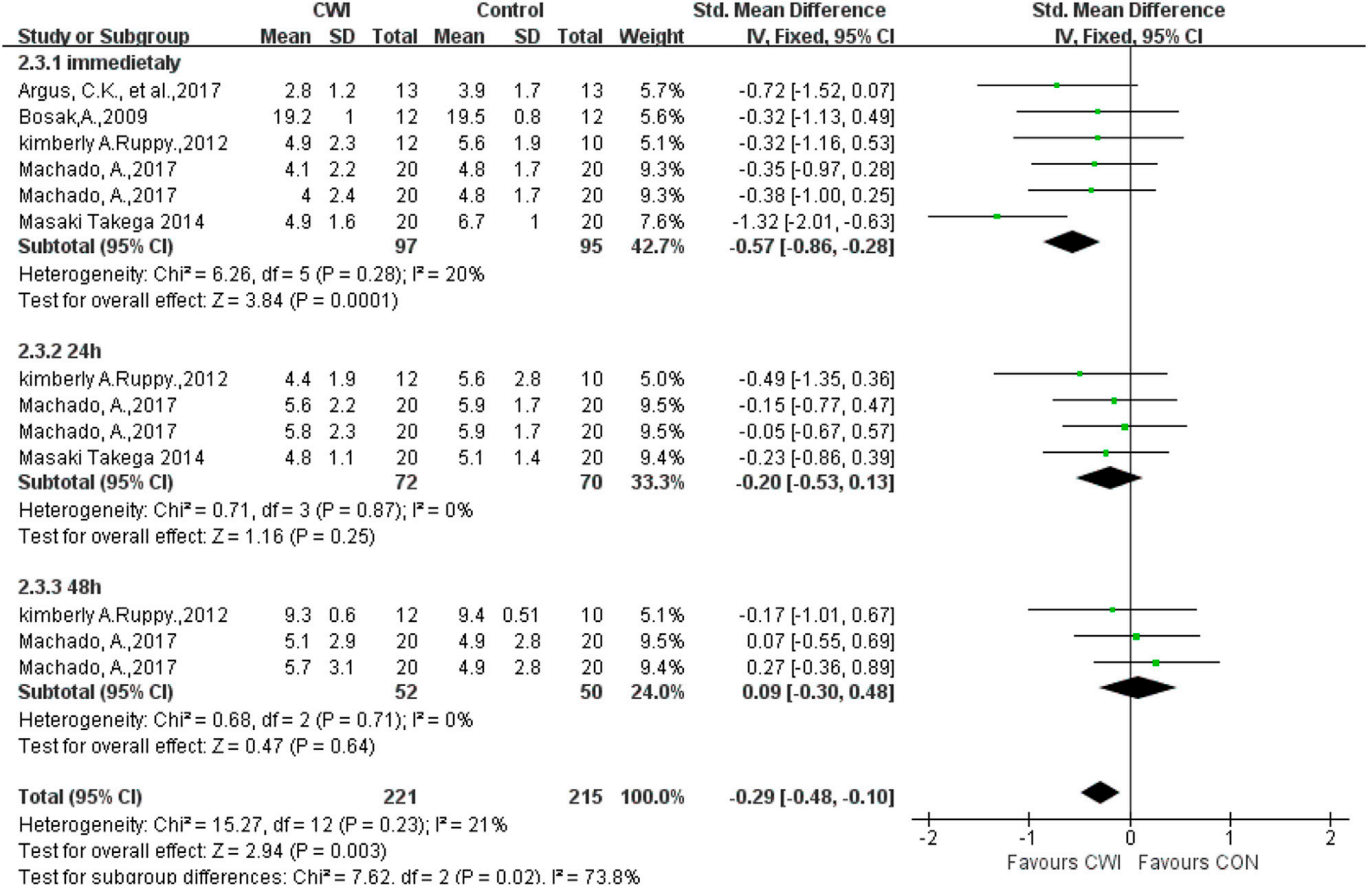
Forest plot of the comparison of CWI versus CON for measurement of RPE. CWI = Cold water immersion, CON = Control, RPE = rate of perceived exertion.

A total of five articles were selected to analyze the RPE in the CWI group versus the CON group. Different measurement tools were chosen for different articles, including 0–10 and 1–10 scales, and hence the SMD was selected (Figure 6). The data showed that there was a significant difference in RPE between the CWI and CON groups at 0 h (SMD -0.57, 95%CL -0.86 to -0.28, 6 trials), but no significant difference was found between the CWI and CON groups at 24 and 48 h (24 h: SMD -0.20, 95%CL -0.53 to 0.13,4 trials); (48 h: SMD 0.09, 95%CL -0.30 to 0.48, 3 trials). Compared with passive recovery, CWI reduced subjective fatigue immediately after long-term exercise but did not help prolonged fatigue.

#### CK

**FIGURE 7:**
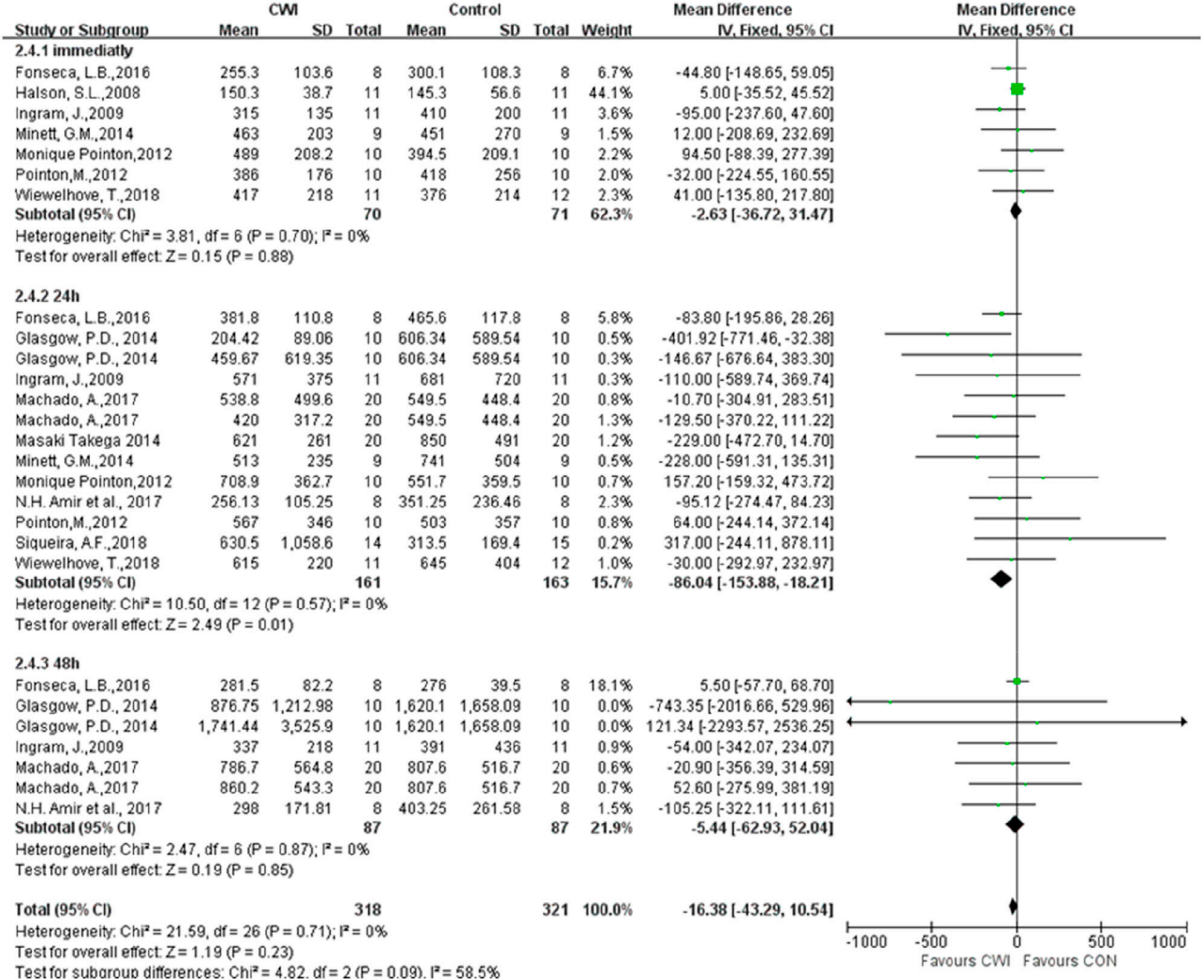
Forest plot of the comparison of CWI versus CON for measurement of CK. CWI = Cold water immersion, CON = Control, CK = creatine kinase.

Twelve articles were selected to determine whether post-exercise CWI affects CK at 0, 24, and 48 h (Figure 7). The results showed a significant difference between the CWI and the CON groups only at 24 h after the experimental intervention (MD -86.04 95% CL -153.88 to -18.21, 13 trials), with CK levels significantly lower at 24 h after the CWI intervention than after passive recovery. In contrast, there was no statistically significant difference between the CWI and CON groups at both 0 and 48 h (0 h: MD -2.63 95%CL -36.72 to -31.47, 7 trails); (48 h: MD -5.44 95%CL -62.93 to 52.04, 7 trials).

#### Lactate

**FIGURE 8:**
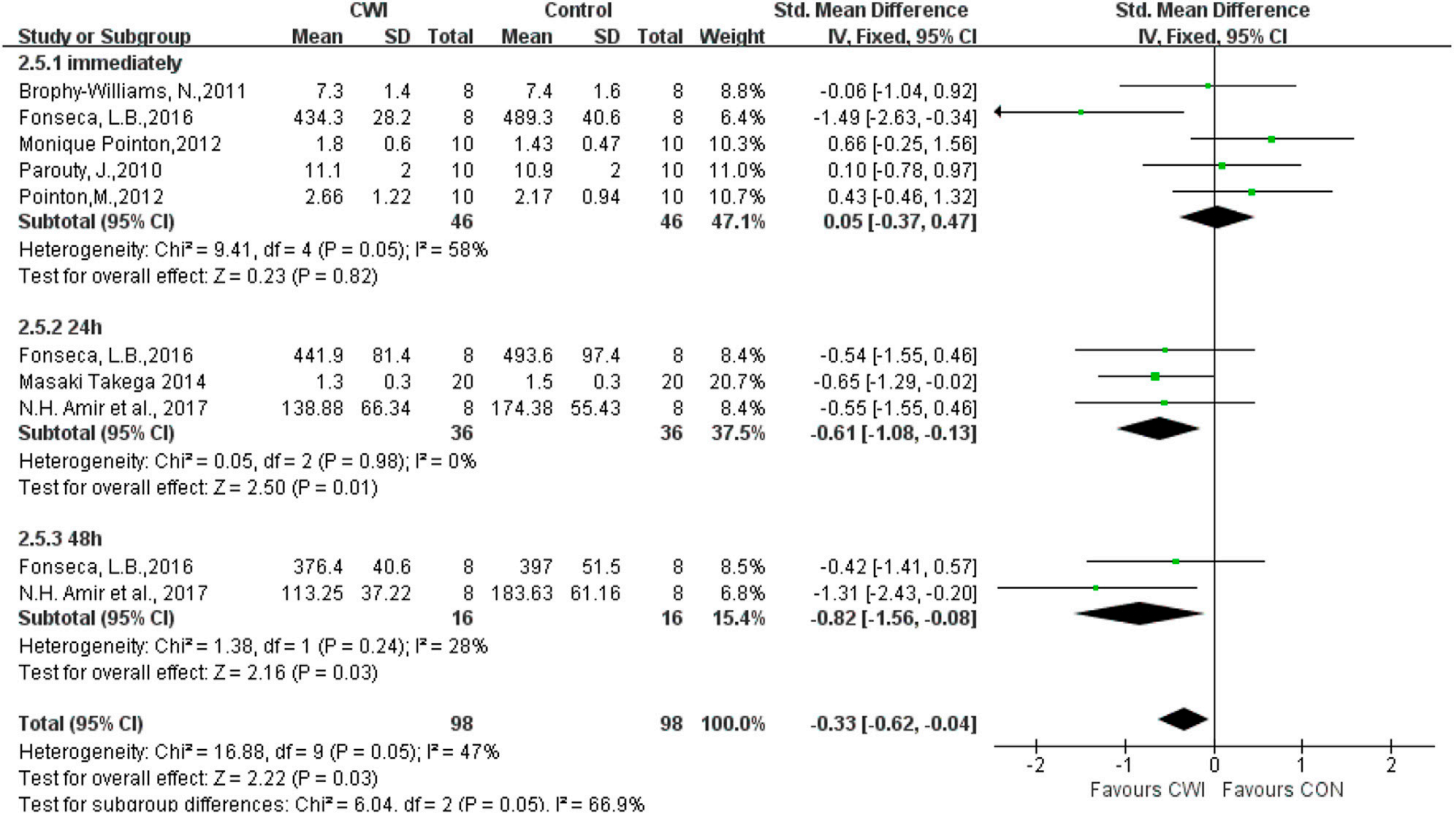
Forest plot of the comparison of CWI versus CON for measurement of Lactate. CWI = Cold water immersion, CON = Control.

Analysis of the effects of post-exercise CWI and passive recovery on lactate revealed significant differences in lactate indicators at 24 and 48 h after intervention (24 h SMD -0.61 95%CL -1.08 to -0.13, 3 trials); (48 h SMD -0.82 95%CL -1.56 to -0.08, two trials) but no significant differences at 0 h (SMD 0.05 95%CL -0.37 to 0.47, 5 trials). Heterogeneity was found among the included literature at 0 h (Chi^2^ = 9.41, df = 4 (*p* = 0.05), I^2^ = 58%), and there was no statistical significance immediately after selecting the random effects model (SMD -0.01 95%CL -0.67 to 0.64, 5 trials) (Figure 8).

A sensitivity analysis was conducted by the one-by-one exclusion method to investigate whether the heterogeneity among studies was caused by individual articles. It was found that heterogeneity decreased when Fonseca et al., 2016 was excluded at 0 h (Chi^2^ = 1.42, df = 3 (*p* = 0.70), I^2^ = 0%), indicating that this article contributes to heterogeneity in present included studies. However, the effect size did not change significantly after exclusion (SMD 0.29 95%CL -0.16 to 0.75, 4 trials), suggesting stable study results.

For purpose of further exploring potential sources of heterogeneity, subgroup analysis was performed based on CWI of different body parts. Heterogeneity was found in the shoulder immersion group at 0 h (Chi^2^ = 5.51, df = 2 (*p* = 0.08), I^2^ = 61%), and then a random effects model was chosen. The results of the shoulder immersion group were not significantly different (SMD -0.43 95%CL -1.35 to 0.49, 3 trials), indicating that CWI to the shoulder is ineffective in reducing exercise-induced lactic acid accumulation immediately. Moreover, there was no heterogeneity in the umbilicus immersion group (Chi^2^ = 0.12, df = 1 (*p* = 0.73), I^2^ = 0%), and thus a fixed-effects model was used to show no significantly different results (SMD 0.54 95%CL -0.09 to 1.18, 2 trials), indicating that CWI to the umbilicus does not change the lactate at 0 h. No significant differences between the two subgroups at 0 h (Test for subgroup differences at 0 h: Chi^2^ = 2.89, df = 1 (*p* = 0.09), I^2^ = 65.4%), indicating that CWI of different body parts does not cause heterogeneity in the lactate group at 0 h.

Subgroup analysis was performed based on the difference in CWI temperature. Heterogeneity was observed in the <10°C group at 0 h (Chi^2^ = 9.35, df = 2(*p* = 0.009), I^2^ = 79%). And therefore a random effects model was selected and the results were not significantly different (SMD -0.08 95%CL -1.30 to 1.13, 3 trials), suggesting that CWI at a temperature <10°C does not affect lactate accumulation at 0 h after intervention. There was no heterogeneity in the ≥10°C group (Chi^2^ = 0.06, df = 1 (*p* = 0.81), I^2^ = 0%), so a fixed effects model was chosen. And the results were not significantly different (SMD 0.03 95%CL -0.63 to 0.68, 2 trials), indicating that water temperature ≥10°C is ineffective in reducing lactate immediately after CWI. There was no significant difference between the two subgroups of CWI at different water temperatures (Test for subgroup differences at 0 h: Chi^2^ = 0.02, df = 1 (*p* = 0.88), I^2^ = 0%), which implied that different CWI temperatures are not a source of heterogeneity in the lactate at 0 h.

#### CRP

**FIGURE 9:**
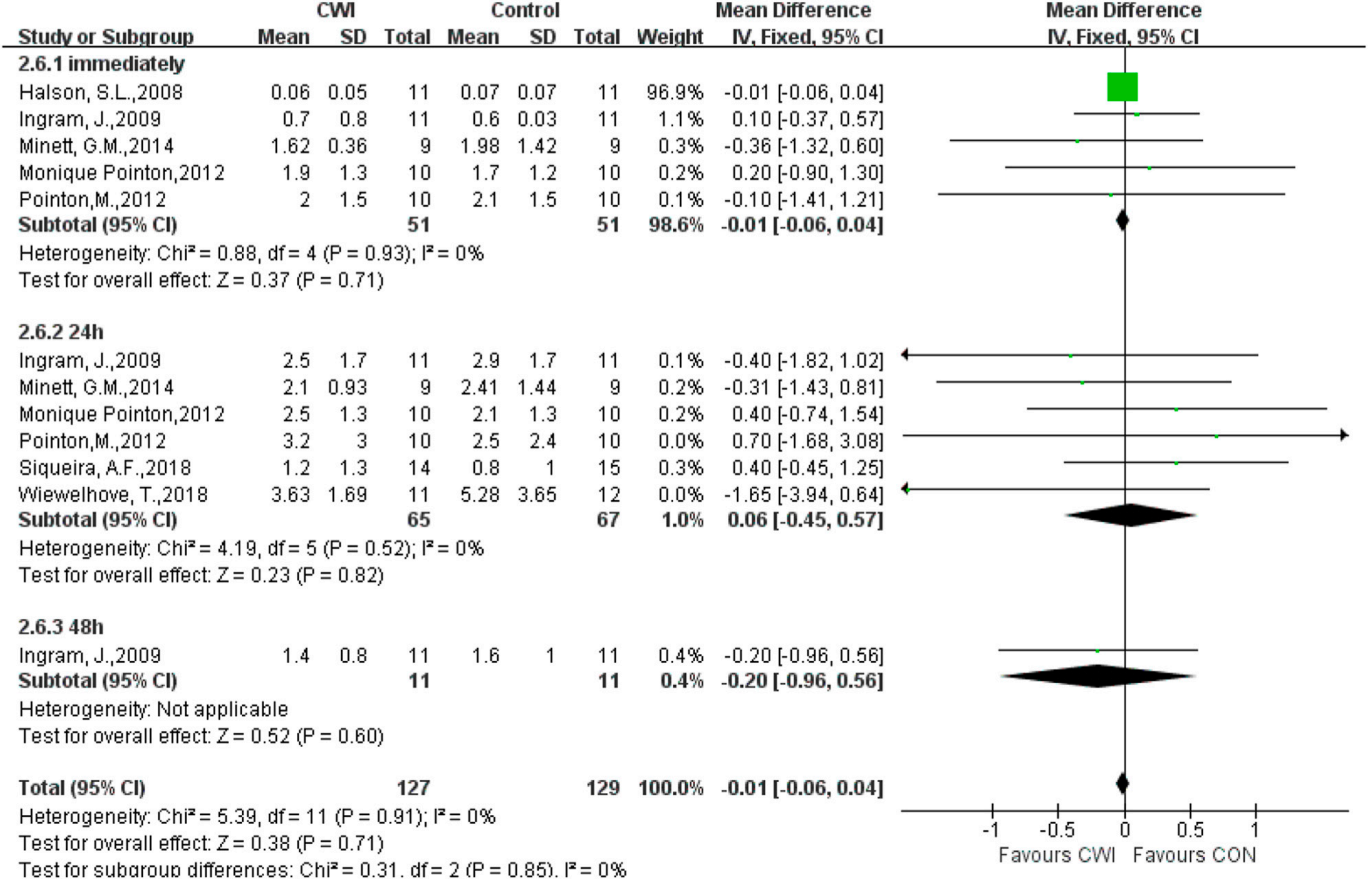
Forest plot of the comparison of CWI versus CON for measurement of CRP. CWI = Cold water immersion, CON = Control, CRP = C-Reactive Protein.

In a total of 7 studies, CRP levels were recorded after the intervention experiment. There was no significant difference in CRP at 0, 24, or 48 h (0 h MD -0.01 95%CL -0.06 to 0.04, 5 trials); (24 h MD 0.06 95%CL -0.45 to 0.57, 6 trials); (48 h MD -0.20 95%CL -0.96 to 0.56, 1trials). Only 1 article was included at 48h, and thus the data at 48 h were ignored (Figure 9).

#### IL-6

**FIGURE 10:**
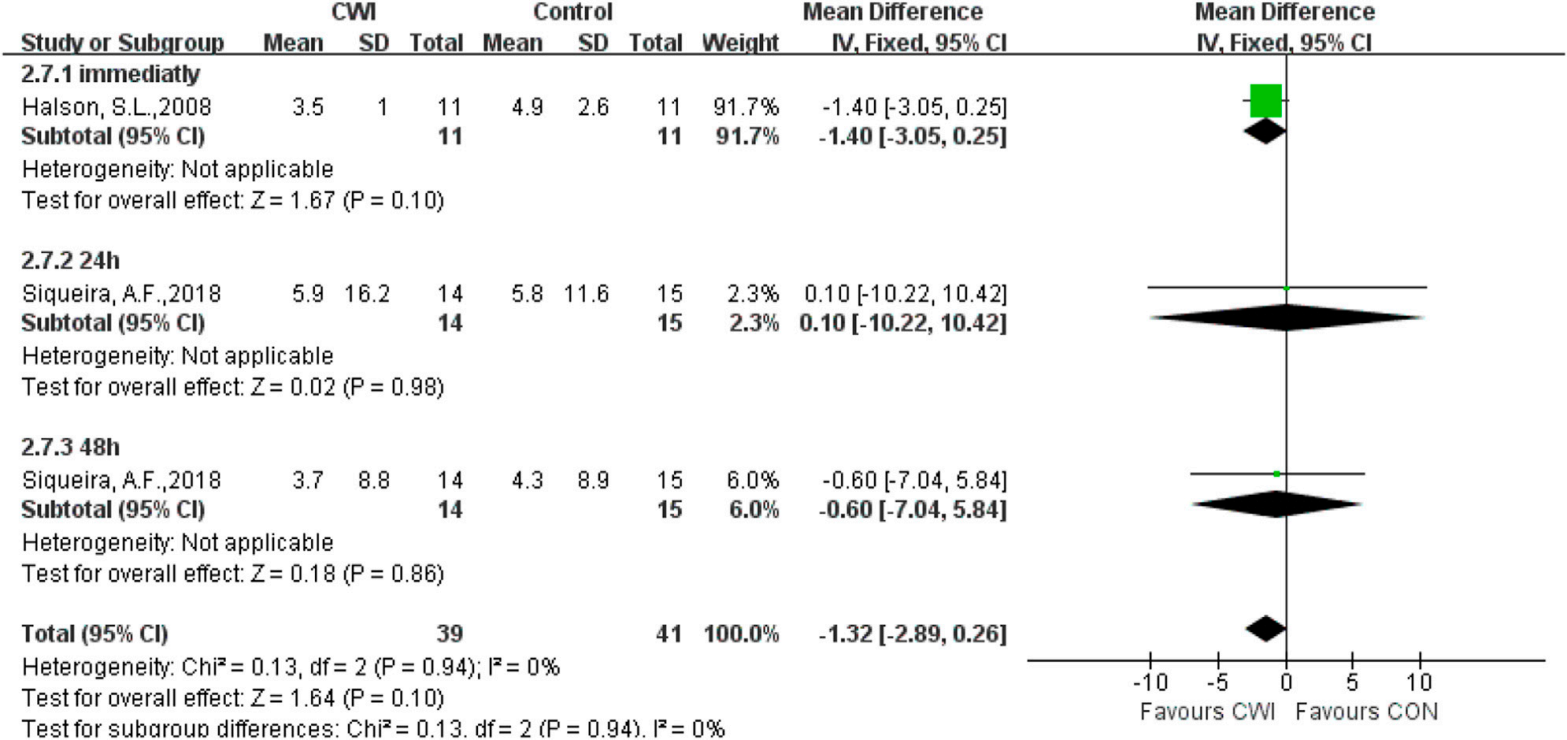
Forest plot of the comparison of CWI versus CON for measurement of IL-6.CWI = Cold water immersion, CON = Control, IL-6 = Interleukin 6.

Only a few articles were selected to analyze IL-6, rendering the results meaningless. Meanwhile, there were no significant differences in inflammatory markers (IL-6), which implied that performing CWI after exercise has no effect on the subsequent physical recovery of the subjects (Figure 10).

## Discussion

### Methodological analysis of included literature

First, the majority of studies had a high or unclear risk of bias, rendering the validity of most results uncertain. The main bias was produced by unblinding in experiments, which is due to the limited possibility of blinding during CWI application. Second, in terms of the allocation concealment procedure, the random allocation of participants was used in only four studies; in two studies, the envelope was used to conceal distribution (Rupp et al., 2012; Glasgow et al., 2014) and in the others two, computers were adopted to ensure random allocation (Machado et al., 2016; Siqueira et al., 2018). Third, random-CON and crossover trials were separately used in different studies of this review. CODs can risk certain carry-over effects between treatment periods that are not present in random-CON designs. In order to improve methodological quality, more randomized controlled trials, allocation concealment, and blind assessors can be included in studies.

### Subjective recovery characteristics

One of the main findings of this review was that post-exercise CWI reduced DOMS and RPE immediately, but with no significant effect on DOMS and RPE at 24 and 48 h (Table 2). It was found by sensitivity analysis that the heterogeneity between DOMS groups might be caused by the study by Ingram, J., in 2009. The reason for this may be the short duration of CWI intervention in this article, which was only 2 min × 5 times with 2.5 min apart. It has been suggested that the short duration of immersion is less effective in relieving exercise-induced muscle (Getto & Golden, 2013).

**TABLE 2:** Summary of results.

	0H	24H	48H
CMJ	↓	— † Temp<10°C ↑; Part —	—
DOMS	↓	— † Temp —; Part —	— † Temp —; Part —
RPE	↓	—	—
CK	—	↓	—
Lactate	— † Temp —; Part —	↓	↓
CRP	—	—	—
IL-6	—	—	—

↓ = significantly decrease in CWI, groups, compared with CON, groups; ↑ = significantly increase in CWI, groups, compared with CON, groups; — = unsignificant difference between CWI, and CON, groups; † = heterogeneity present between CWI, and CON, groups; Temp = CWI, temperatures; Part = CWI, of different body parts; CMJ , countermovement jump; DOMS, delayed-oneset muscle soreness; RPE , rating of perceived exertion; CK , creatine kinase; CRP = C-Reactive protein; IL-6 , Interleukin 6.

The physiological mechanisms by which CWI performance after high-intensity exercise can immediately reduce DOMS and RPE may be explained as follows. 1. CWI can reduce edema, pain, and metabolite accumulation by lowering body temperature and regulating central nervous system mechanisms (Vaile et al., 2011; Leeder et al., 2012). Immersion in cold water results in a decline in acetylcholine production, which in turn causes a decrease in neuronal transmission rate and the ability to reduce muscle spasm. Also, a 10°C–13°C decrease in the skin temperature can reduce nerve conduction velocity by 10%–33% (Algafly & George, 2007). 2. CWI causes vasoconstriction due to hydrostatic pressure, which increases venous blood return, thus reducing edema and pain, and accelerating metabolic elimination (Wilcock et al., 2006). 3. CWI has been reported to reduce lymphatic and capillary permeability (Wilcock et al., 2006), thereby reducing fluid diffusion, which may help reduce exercise-induced inflammation, as well as edema and pain (Wilcock et al., 2006). 4. CWI can provide a placebo effect on subjects to some extent. Double-blind bias during the experiment can affect the judgment of the subjects, causing a placebo effect.

### Objective recovery characteristics

The main conclusion of this review is that CWI reduces CMJ at 0h, CK at 24h, and lactate at 24h and 48 h after intervention. However, post-exercise CWI does not produce any effect on CRP or IL-6 (Table 2). It was found by sensitivity analysis that the heterogeneity between CMJ studies may be caused by the study of Fonseca, L.B., in which the CWI group was immersed in cold water at 6°C ± 0.5°C for 16 min. One study indicated that immersion in cold water at 11°C–15°C for 11–15 min is the most appropriate and beneficial for fatigue recovery after exercise (Machado et al., 2016). In this review, studies involving a CWI group at 11°C–15°C were mainly selected. Hence, the great difference in water temperature between the study by Fonseca and the others may be the reason for the heterogeneity between them.

This review suggests that CMJ immediately decrease after CWI, which is consistent with the findings from several studies (Fischer et al., 2009; Nuhmani et al., 2012). The evaluation of the effect of cryotherapy on physical performance showed a 17.5% decline in vertical jump performance immediately after CWI (Patterson et al., 2008). A 37% reduction was observed in vertical jumps immediately when the objects underwent CWI (Didehdar and Sobhani, 2019). The jump height was discovered to decrease by 4.2% for every 1°C decrease in muscle temperature (Bergh & Ekblom, 1979). The reason is that cold muscles can result in lower nerve impulse frequency and longer relaxation time.

The reasons for the decrease in CMJ performance immediately caused by CWI are as follows. 1. CWI decreased the skeletal muscle temperature, resulting in an increase in skeletal muscle viscosity. The CMJ is a plyometric contraction exercise. When the muscle is elongated, elastic potential energy and stretch reflex contraction will be generated; when the elastic potential energy, stretch reflex contraction, and active concentric contraction form a resultant force, the muscle will produce a larger contraction force. Increasing the muscle viscosity can decrease muscle extension and elasticity, and impair elastic potential energy, which ultimately causes a decrease in CMJ performance. 2. CWI affects the exchange between Ca_2_+ and Na_2_+ in nerve cells, which may lead to a delay in action potential generation, contraction velocity, and force-producing capacity, thus reducing dynamic contraction force and the subsequent motor performance (Machado et al., 2016). Meanwhile, studies have shown a linear relationship among muscle temperature, nerve conduction rate, and muscle activation through electromyography (EMG) recordings. When the muscle temperature is low, the nerve conduction rate decreases, and the central nervous system becomes less excitable (Machado et al., 2017), with the maximum muscle strength declining.

Post-exercise CWI could decrease CK levels at 24 h. These results are similar to the findings of the study by Ascensao et al., in which it was concluded that performing CWI for athletes at 10°C for 10 min after a simulated soccer match reduces serum CK levels at 24 h (Ascensao et al., 2011). Also, they are similar to the results of the other meta-analyses (Higgins et al., 2017). CWI could reduce lactate levels at 24 and 48 h. However, CWI did not affect CRP or IL-6. An inverse relationship was observed between serum levels of LDH, with muscle soreness perceived (Júnior et al., 2014), which was consistent with the results of this review. Lactate levels did not change significantly but DOMS levels significantly decreased from 0 h by CWI application after exercise, compared with passive recovery. However, while CWI significantly decreased lactate levels, it did not change DOMS levels at 24 and 48 h after exercise.

As previously stated, local cooling may change the skeletal blood flow by reducing the capillary permeability (Eston & Peters, 1999; Vaile et al., 2011). Also, it may decrease the passive leakage of intracellular substances utilized as subliminal indicators of muscle injury, such as decreased CK efflux. Prolonged analgesia may result from intravascular fluid shifts due to vasoconstriction, thus facilitating nutrient and waste transport, as well as reducing muscle edema (Cheung et al., 2003; Ihsan et al., 2013; Yanagisawa et al., 2014). In addition, there were inadequate data on the other biochemical indicators such as CRP and IL-6, and thus meta-analyses were difficult to conduct.

### Subgroup analysis characteristics

Differences in body parts and the water temperature in CWI application did not affect subjective fatigue recovery, nor did they affect objective recovery. This was different from the study result by Poppendieck W (Poppendieck et al., 2013), indicating that whole-body CWI is more effective than partial-body cooling. The reason is that whole-body CWI reduces the core body temperature effectively. Moreover, whole-body cooling is subjected to higher hydrostatic pressure than partial-body cooling.

### Limitations

Limitations in study designs were also identified. First, we have chosen articles from 2002 to 2022, but in fact articles just to 2018. Second, blinding of participants in studies was problematic, especially with hydrotherapy, which left them vulnerable to the placebo effect. Thirdly, the subgroup analysis was not comprehensive. The subgroup analysis was based solely on different body parts that underwent CWI, and the different temperatures. There was a lack of comparisons of differences between eccentric and high-intensity exercise and between genders. Fourthly, grouping together the active population, recreational athletes and professional athletes limits the applicability of this present findings.

## Conclusion

CWI is commonly used as a fatigue-recovery measure after exercise. In this review, the effect of CWI on post-exercise fatigue recovery was systematically analyzed. Subjective sensations seemed to recover immediately, as did objective biochemical markers such as CK and lactate after 24 h. CWL of different body parts and different water temperatures have no effect on fatigue recovery.

In future, a randomized controlled study design needs to be adopted as much as possible in studies to improve the methodological quality. Also, a double-blind design should be conducted for subjects and experimenters whenever possible to ensure that they are not affected by each other’s placebo. Additionally, it is necessary to perform multidimensional subgroup analyses such as different gender and exercise intensity. Furthermore, the dose of CWI should be reconsidered.

## Data Availability

The original contributions presented in the study are included in the article/supplementary material, further inquiries can be directed to the corresponding author.
